# The developmental trajectory and influencing factors of school refusal behavior among Chinese adolescents

**DOI:** 10.1186/s40359-026-04859-x

**Published:** 2026-05-28

**Authors:** Rong-Man Yuan, Na Fu

**Affiliations:** 1 Students’ Mental Health Center, Beijing Youth Politics College, Beijing, 100102 China; 2 Faculty of Education, Beijing Normal University, Beijing, 100875 China

**Keywords:** School refusal behavior, Developmental trajectory, LGMM, Chinese adolescents

## Abstract

**Background:**

The developmental trajectory among Chinese adolescents remains unclear. This study aimed to explore the developmental trajectory of school refusal behavior and examine how individual and environmental factors predict this trajectory among Chinese adolescents.

**Participants and methods:**

A longitudinal design was used with 1,308 middle and high school students (*mean*_age_ ± *SD*_age_ = 14.35 ± 1.50, 41.36% boys) who were followed up across three time points (T1, T2, and T3) over one year.

**Results:**

The latent growth mixture model analysis (LGMM) revealed four subgroups within the developmental trajectory of school refusal behavior among Chinese adolescents: a low-decrease group (*n* = 678), a high-stable group (*n* = 445), a high-decrease group (*n* = 104), and a low-increase group (*n* = 81). Furthermore, group membership was predicted by academic resilience, family functioning, and class interpersonal harmony.

**Conclusions:**

These findings suggest that effective interventions are needed to address the various developmental trajectories of school refusal behavior in adolescents.

## Introduction

School refusal behavior, a subset of broader school attendance issues, is a significant concern affecting roughly 1 ~ 15% of adolescents worldwide [1, 2]. It has been found that approximately 28% of students may experience school refusal behavior at some stage during their schooling [3–5]. School refusal behavior is a broad term that encompasses concepts such as truancy, school refusal, and school phobia [3]. Specifically, it refers to the emotional and behavioral challenges youth face in attending or remaining in school [3]. Studies have shown that school refusal behavior negatively impacts adolescents’ academic performance and daily life in both the short and long term [2, 6–10]. School refusal behavior can occur during the school-age stage [10]. Still, it is most prevalent during adolescence, a phase characterized by significant physiological, psychological, and social pressures [11].

Negative consequences of school refusal behavior include low academic performance [12], difficulties with emotional adjustment [13], and an increased risk of behavioral problems, substance use, and sleep issues [14, 15]. School refusal behavior is also associated with school dropout and conditions like anxiety disorders and antisocial behavior [16, 17]. Given the significance of these findings, systematic research on school refusal behavior in adolescents has important theoretical and practical implications. Previous research, using cross-sectional surveys, has focused on individual differences in the functional profiles of school refusal behavior [18]. Yet, it has largely overlooked the differences in the dynamic developmental trajectories of school refusal behavior in adolescents over time, as examined through longitudinal research. To address this gap, it is crucial to explore the developmental trajectories of school refusal behavior and identify associated protective factors. Such exploration will advance understanding of the behavior’s progression and inform the development of more precise and effective interventions.

School refusal behaviors, a subset of school attendance issues, exhibit distinct patterns across different educational stages. They tend to decrease during early elementary school, increase sharply in middle school, and continue to rise through high school, peaking in the final year [19, 20]. However, research on the developmental trajectory of school refusal behaviors remains limited, and existing studies on the developmental trajectory of school attendance have produced inconsistent conclusions. Several studies have examined the developmental trajectories of school attendance and absenteeism among diverse student populations, consistently identifying multiple distinct patterns. For example, Benner and Wang found that attendance declined from middle to high school, with subgroups showing stable high, high-decreasing, mid-decreasing, and low-decreasing patterns [21]. Dräger and colleagues (2023, 2024) identified five trajectories ranging from consistently low absences to strongly increasing authorized or unauthorized absences [22, 23]. Similarly, Tunkkari et al. reported low-stable, high-stable, increasing, and early-started absence trajectories among Finnish adolescents [24]. Only one study directly examined the developmental trajectories of school refusal behaviors using retrospective parent reports of school attendance over three consecutive years. The research identified five distinct trajectories: for children, “beaded absences” and “rapid recovery”; for adolescents, “prolonged recovery”, “gradual decline”, and “rapid decline” [25].

Although school refusal behavior has received increasing attention, its developmental trajectories remain relatively under-examined compared with the sizeable body of longitudinal work on school attendance. Moreover, attendance-focused studies have predominantly relied on administrative absence counts that cannot distinguish emotionally driven refusal from other forms of non-attendance. This conflation masks heterogeneity in how refusal behaviors evolve during adolescence—a developmental period characterized by increasing academic and social pressures. To date, however, few studies have systematically examined the latent profile characteristics of school refusal behavior among Chinese adolescents. Given China’s specific educational ecology, culture-specific classification research is imperative, leaving ample room for further exploration in this field.

There are theoretical and empirical reasons to expect that school refusal behavior does not follow a single, uniform developmental pathway. From a developmental psychopathology perspective, the principle of multifinality [26] holds that the same initial behavioral or risk condition can lead to qualitatively different developmental outcomes, depending on the interplay of risk and protective factors across time and context. Applied to school refusal, this principle suggests that while some adolescents may exhibit stable low levels of refusal over time, others may follow chronic high trajectories, and still others may show increasing or decreasing patterns. In other words, the developmental course of school refusal is inherently heterogeneous rather than homogeneous. Empirically, the person-centered research on school absenteeism reviewed above has consistently identified multiple distinct trajectories [21–24], ranging from consistently low absence to chronic high absence, as well as increasing and decreasing patterns. Given that school refusal behavior is a more complex and emotionally driven subset of attendance problems [4], it is reasonable to expect that its development would exhibit at least comparable, if not greater, heterogeneity. Identifying these distinct trajectories is a necessary first step toward understanding their differential predictors and developing targeted interventions.

Although research has identified a wide range of factors associated with school refusal behavior—spanning individual psychological, family, and school-related domains [10, 27–31]—most of these studies have treated school refusal as a unitary phenomenon, examining correlates of average levels of refusal without distinguishing between potentially distinct developmental trajectories. Consequently, it remains unclear whether the same set of factors uniformly characterizes all adolescents with school refusal or whether their effects differ across distinct developmental trajectories.

Identifying trajectory-specific predictors is essential for moving from generic intervention recommendations to precision prevention strategies tailored to the needs of different subgroups.

To provide a coherent framework for selecting predictors and understanding their differential effects across trajectories, we integrate Bronfenbrenner’s bioecological model [32] with Hobfoll’s Conservation of Resources (COR) theory [33]. Bronfenbrenner’s model [32] conceptualizes human development as shaped by the interactions between a developing individual and their nested ecological contexts. According to this framework, the microsystem—the immediate environment containing the developing person (e.g., family, classroom)—exerts the most direct influence on day-to-day behavior [32, 34, 35]. For adolescents, the family and the school classroom represent the two most salient microsystems. However, Bronfenbrenner’s model also emphasizes that development is driven by proximal processes occurring between the individual and their environment [32]. This means that both individual characteristics (e.g., personal resources) and microsystem characteristics (e.g., relational quality) jointly shape developmental outcomes.

Guided by this integrated framework, we identified three key resources representing different ecological levels: academic resilience, an individual-level personal resource for managing academic stress; family functioning, a relational resource within the family microsystem; and class interpersonal harmony, a contextual resource within the school microsystem. According to COR theory, deficiencies in these resources—whether at the individual or environmental level— increase adolescents’ vulnerability to maladaptive developmental trajectories of school refusal.

Among the individual psychological factors, academic resilience is particularly significant. Defined as the tendency to maintain academic stability and achieve success despite challenging social and psychological stressors [36–38], it plays a crucial role in understanding the development of school refusal behavior. Students with high academic resilience are better equipped to maintain their academic performance and psychological well-being in the face of adversity, thereby significantly reducing the likelihood of school refusal [36, 39]. Ingul and Nordahl (2013) found that academic resilience is essential for reducing school dropout rates among young people [40]. Recent studies have also highlighted the potential of academic resilience as a protective factor that can mitigate several risk factors associated with school refusal [16]. From a trajectory perspective, students with low academic resilience may lack the internal resources needed to cope with accumulating academic stress, making them more likely to follow chronic or escalating refusal pathways, whereas those with high resilience are better positioned to maintain low or decreasing trajectories.

From the perspective of ecological systems theory [32], the family, as a proximal context, may directly influence adolescents’ school refusal behavior. Family functioning, which refers to a family’s capacity to maintain emotional bonds among members and effectively address family challenges [41], is essential for understanding these correlations. In this context, children’s perceptions of family harmony and family functioning serve as protective factors against school refusal behavior. Previous studies have identified a close association between various family-related factors and school refusal behavior in adolescents. Children’s perceptions of family harmony serve as a protective factor, while family conflict, dysfunctional environments, and poor communication increase risk [2, 42–44]. A meta-analysis confirmed that family functioning is significantly lower in families of children who refuse school [1]. From a trajectory perspective, adolescents from poorly functioning families lack the external scaffolding needed to cope with school-related stress. Without this relational resource at home, they may be more vulnerable to following stable, high-level refusal trajectories, whereas those with supportive families are better equipped to navigate difficulties and thus more likely to belong to adaptive or recovering trajectory groups.

The class, as a key microsystem within the school environment, serves as a primary source of children’s interpersonal relationships and activities and significantly influences their mental health [34, 35]. In the Chinese educational context, where students spend most of their school time with the same classmates and the class is often considered “the second home” [45–47], the quality of classroom interpersonal relationships is particularly salient. The concept of class interpersonal harmony captures this quality through three sub-dimensions: teacher-student climate, peer climate, and class structure [48]. Previous research has shown that positive teacher-student and peer relationships correlate with lower internalizing and externalizing symptoms [49–51], whereas disharmonious relationships increase the risk of behavioral problems [52–54]. Given that such problems are closely linked to school refusal [55], class interpersonal harmony likely plays a protective role. From a trajectory perspective, students who perceive their classroom as disharmonious experience school as an aversive environment, increasing their likelihood of following escalating or chronic refusal trajectories. Conversely, those who experience harmony are more likely to maintain low levels of refusal or show recovery over time, as the classroom itself serves as a source of support rather than stress.

### Present study

This study aimed to fill this gap by exploring the heterogeneous trajectories of school refusal behavior among adolescents and by identifying longitudinal clusters associated with predictors. We employ the latent growth mixture model (LGMM) to investigate whether there are inherent group differences in the developmental trajectories of school refusal behavior among adolescents, and to study how individual and environmental factors influence these trajectories.

## Methods

### Participants and procedure

This study was approved by the Ethics Committee of Beijing Normal University (IRB ID no. BNU202311010167). This study employed a convenience sampling method, recruiting participants from three public middle schools in Beijing (one school) and Shenzhen (two schools), China. Participants received the written instructions and were informed about the nature of the study and the confidentiality of their responses. The research assistants (trained postgraduate students) read the instructions aloud and ensured that each participant understood them. Students were assessed in their classrooms during school hours and were informed that they could withdraw from the study at any time. Respondents took approximately 15 min to complete the surveys. Follow-up surveys were administered in January 2024, July 2024, and January 2025. In January 2024, 1317 students participated in the first wave of measurement, of whom 1310 also participated in the follow-up wave conducted in July. Thus, the final sample comprised 1308 students who completed measurements at three time points. Among the sample, 541 individuals were male, and 767 were female; ages ranged from 10 to 18 years, with a mean age of 14.35 ± 1.50 years. The study comprised 257 Grade 7 students, 550 Grade 8 students, 260 Grade 9 students, and 241 Grade 10 students.

### Measures

We operationalized “individual and environmental factors” through three specific constructs grounded in ecological systems theory. Academic resilience represents the individual level—intrapersonal resources for managing academic stress. Family functioning (APGAR) captures the microsystem of the family—emotional bonds and problem-solving capacity in the home environment. Class interpersonal harmony captures a distinct microsystem of the school—teacher-student relationships, peer climate, and classroom structure.

#### School refusal behavior

School refusal behavior was assessed using the Chinese version of the School Refusal Assessment Scale-Revised. The original version of the SRAS-R is a 24-item self-report measure that assesses the four functional conditions for the maintenance of school refusal behavior [56]. This study employed a revised 32-item version of the Chinese SRAS-R, which includes five dimensions: avoidance of distressing school-related stimuli (8 items), fear of negative perception by teachers and peers (6 items), strong need for parental attention (5 items), tendency to engage in more appealing out-of-school activities (6 items), and school refusal behavior manifestations (7 items). Each item was rated on a five-point Likert scale (0 = strongly disagree to 5 = strongly agree), with higher scores reflecting greater school refusal behavior. The Cronbach’s alpha coefficients for the dimensions were 0.93, 0.90, 0.88, 0.90, and 0.92, respectively, and the overall Cronbach’s alpha was 0.94.

#### Academic resilience

Academic resilience was assessed using a four-item scale (“I think I’m good at dealing with schoolwork pressures”) developed by Martin and Marsh [57]. The Chinese revised version of the scale was used in this research [58]. Students rated each item on a 5-point Likert scale, ranging from 1 (“strongly disagree”) to 5 (“strongly agree”). The academic resilience score was calculated as the average of responses to scale items, with higher scores indicating stronger academic resilience. The Cronbach’s alpha coefficient of the scale was 0.90.

#### Family functioning

Family functioning was assessed using the Family APGAR (Adaptation, Partnership, Growth, Affection, Resolve) Questionnaire, developed by Smilkstein [59]. The Chinese version of the scale, adapted by Lv [60], was employed in this study. The questions (items) in the Family APGAR are designed to permit qualitative measurement of family members’ satisfaction with five components of family function: Adaptation, Partnership, Growth, Affection, and Resolve. Each of the five items is scored on a three-point scale: 0 = hardly ever,1 = some of the time, and 2 = almost always. The higher the total score is, the better the respondent’s perceived family functioning. The Cronbach’s _α_ of the family functioning in this study was 0.90.

#### Class interpersonal harmony

The Student Perceived Class Interpersonal Harmony Questionnaire, developed by Chen and Li [48], was used to examine students’ perceptions of interpersonal harmony within their classrooms. The questionnaire includes 20 questions across three dimensions: teacher interpersonal climate (7 items), peer interpersonal climate (6 items), and class structure (7 items). The questionnaire was scored on a 5-point Likert scale, ranging from 1 (“never like this”) to 5 (“always like this”). Higher scores indicate greater perceived interpersonal harmony within the class. It has demonstrated good reliability and validity in the Chinese cultural context [45, 61]. In this study, the internal consistency coefficients of teacher interpersonal climate, peer interpersonal climate, and class structure were 0.85, 0.86, and 0.81, respectively, and the overall Cronbach’s alpha was 0.82.

### Statistical analysis

Analyses were conducted via Mplus 8.3 and SPSS 29.0. First, descriptive and correlational analyses were conducted on the major variables. Second, we estimated the heterogeneity in the development of school refusal behavior among adolescents using the LGMM [62]. Longitudinal research frequently encounters population heterogeneity, in which individuals follow distinct developmental trajectories that cannot be adequately captured by a single average trajectory. This approach is known as the LGMM [62]. The data analysis chiefly consisted of two steps. The first step was to analyze whether heterogeneity in adolescents’ developmental trajectories was reflected in differences among latent classes. In this step, starting with the initial model (if the entire sample belongs to a single category), the number of model categories was gradually increased until the best-fitting model was identified. In the second step, a multiple logistic regression model was developed. This model used the latent-class classification results from the initial step as dependent variables and incorporated individual and environmental factors as independent variables. The goal of this step was to examine how individual and environmental factors influence the latent classes in the developmental trajectories of school refusal behavior among adolescents.

In this study, the log-likelihood, Akaike information criterion (AIC), and sample-size-adjusted Bayesian information criterion (aBIC) were the primary indicators used to assess the fit of the LGMM model. Lower values of these indicators signify a better model fit [63]. In latent class analysis, entropy is commonly used to evaluate classification accuracy. An entropy value between 0 and 1 indicates classification precision; an entropy of 0.6 suggests approximately 20% classification errors, whereas a value near 0.8 implies over 90% accuracy [63]. Additionally, Mplus offers the Lo-Mendell-Rubin (LMR) likelihood ratio test and the bootstrap likelihood ratio test (BLRT) to compare the fit of different latent class models. Significant *p*-values for these tests indicate that a k-class model fits the data significantly better than a k-1-class model [63].

## Results

### Descriptive statistics

Table 1 shows the mean levels of school refusal behavior, the standard deviations, and the correlation coefficient matrix for each survey application. The results show that the mean school refusal behavior across the three surveys varied very little, suggesting that the trend in mean school refusal behavior was relatively stable.

**Table 1:** Descriptive statistics and correlation coefficient matrix

Model	T1	T2	T3
1.T1	—		
2.T2	0.72^**^	—	
3.T3	0.70^**^	0.74^**^	—
*M*	2.19	2.11	2.08
*SD*	0.89	0.81	0.87

T1 = first survey; T2 = second survey; T3 = third survey

^**^p < 0.01

### Results of the latent growth mixture model

#### Latent growth mixture model fit information

This study developed LGMMs with 1–5 latent classes, and their fit indices are summarized in Table 1. The number of classes in mixture modeling is determined using criteria such as AIC, BIC, aBIC, classification accuracy index, entropy, BLRT, and LMR (Lo-Mendell-Rubin, LMR) [64]. The indices favored the four-class model in this study. Although the entropy value in the three-group model was better than in the four-group model, the other criteria (AIC, BIC, aBIC) showed that the four-class model offered a better fit. Additionally, the latent class proportion should not be less than 5% [65]. However, the minimum proportion for the latent class was 4% in the three-class model, indicating that one of the three classes was not meaningful. Therefore, based on the convergence of multiple statistical indicators—including superior information criteria (lower AIC, BIC, and aBIC), significant likelihood ratio tests (BLRT and LMR), acceptable classification quality (entropy > 0.80), and adequate class sizes—coupled with theoretical interpretability (the 4-class solution captures an escalating trajectory invisible in the 3-class model), the four-class model was selected as the final unconditional model. The model fit indices are illustrated in Table 2. Based on the above findings, we applied a classification scheme with four latent classes (C1, C2, C3, and C4); the attribution probability array for these four latent classes is shown in Table 3. Table 3 shows that participants (columns) in each class had average probabilities (rows) of attribution to each latent class ranging from 80.1% to 91.3%. This finding suggests that the classification model’s results, which employed four latent classes, were credible.

**Table 2:** Summary of LGMM fit information

Model	K	Log(L)	AIC	BIC	aBIC	Entropy	LMR(*p* value)	BLRT(*p* value)	Class probability
1	8	−3890.003	7796.005	7837.415	7812.003				
2	11	−3814.057	7650.115	7707.054	7672.112	0.733	*p* < 0.001^***^	*p* < 0.001^***^	0.60/0.40
3	14	−3774.519	7577.039	7649.506	7605.035	0.816	*p* < 0.05^*^	*p* < 0.001^***^	0.04/0.41/0.55
4	17	−3719.888	7473.775	7561.772	7507.771	0.794	*p* < 0.05^*^	*p* < 0.01^**^	0.09/0.07/0.33/0.51
5	20	−3697.998	7435.997	7539.522	7475.991	0.768	*P* = 0.2385	*P* = 0.2244	0.29/0.12/0.45/0.05/0.09

*K* Number of Free Parameters, *Log(L)* Loglikelihood, *AIC* Akaike information criterion, *aBIC* Sample-Size Adjusted BIC, *LMR* Lo-Mendell-Rubin Adjusted LRT, *BLRT* Bootstrapped Likelihood Ration test

^*^p < 0.05

^**^p < 0.01

^***^p < 0.001

**Table 3:** Average attribution probability (rows) of participants in each latent class (columns

	C1 (%)	C2 (%)	C3(%)	C4 (%)
C1	86.5%	0.0%	4.8%	8.7%
C2	0.0%	80.1%	13.2%	6.6%
C3	2.3%	3.5%	86.6%	7.5%
C4	2.3%	1.0%	5.4%	91.3%

#### Developmental trajectories of school refusal behavior in each latent class

Based on the finding that a model with four latent classes provided the best fit, we then examined the developmental trajectories within each latent class. The mean intercept (α) and slope (β) could be obtained for each latent class in the LGMM; specifically, the mean intercepts of the latent classes were C1: 1.581 (*SE* = 0.031,* t* = 50.870, *p* < 0.01); C2: 2.939 (*SE* = 0.055, *t* = 53.200, *p* < 0.001); C3:3.027 (*SE* = 0.085, *t* = 35.731, *p* < 0.001); and C4: 1.849 (*SE* = 0.100, *t* = 18.437, *p* < 0.001).

**Fig. 1:**
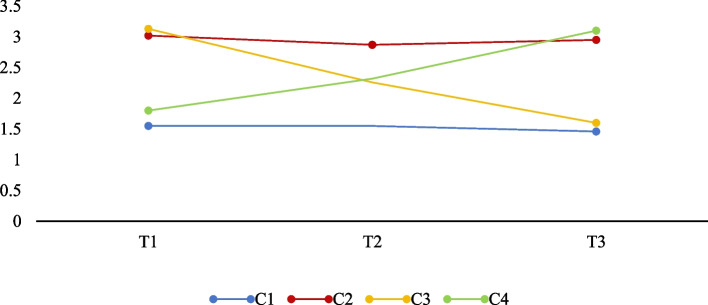
Four groups of different school refusal behavior developmental trajectories. Note: the y-axis is the mean score of school refusal behavior; the x-axis is the survey waves (T1 January 2024, T2 July 2024, T3 January 2025. The blue line represents the C1 class (the low-decrease group); the red line represents the C2 class (the high-stable group); the yellow line represents the C3 class (the high-decrease group), and the green line represents the C4 class (the low-increase group)

Based on the sample means, the development trends for each category are shown in Fig. 1. As shown in Fig. 1, individuals within the four groups showed different growth patterns. Most participants (51.8%, *n* = 678) were distributed in the class with the lowest initial scores and maintained a decreased level thereafter (an estimated mean slope B of −0.044, *p* < 0.001). This trajectory was named the “low-decrease group (C1)”. The second trajectory (34.0%, *n* = 445), labeled the “high-stable group (C2)”, began with a high initial score, then experienced a slight decrease, followed by a minimal increase. Despite these minor fluctuations, the group demonstrated an overall pattern of relative stability over the three time points (an estimated mean slope B of −0.029, *p* < 0.001). The third trajectory (8.0%, *n* = 104) had the highest initial score and showed a sharp decrease to a low level of school refusal behavior (an estimated mean slope B of −0.704, *p* < 0.001); thus, it was named the “high-decrease group (C3)”. The fourth trajectory (6.2%, *n* = 81) was defined as the “low-increase group(C4)”, starting from relatively low initial scores and progressing to the highest scores over time (an estimated mean slope B of 0.602, *p* < 0.001).

#### Predictors of school refusal behavior developmental trajectories

To determine the effects of individual and environmental factors on the developmental trajectories of school refusal behavior among adolescents, these variables were introduced into the LGMM model. In logistic regression, individual factors (academic resilience) and environmental factors (family functioning and class interpersonal harmony) served as predictive variables, while the latent class classification results served as the dependent variable. This procedure yielded odds ratio (OR) coefficients, which reflected the effects of individual and environmental factors among adolescents in the different groups.

The results of logistic regression predicting classes are shown in Table 4. Regarding the academic resilience effect, compared to the low-decrease group (C1), the high-stable group (C2) had an OR value of 0.552 (*p* < 0.001), and the high-decrease group (C3) had an OR value of 0.535 (*p* < 0.001). Compared to the low-increase group (C4), C2 had an OR value of 0.632 (*p* = 0.002), and C3 had an OR value of 0.612 (*p* = 0.005). These results indicated that students with greater academic resilience were less likely to be in the high-stable group (C2) or the high-decrease group (C3) than in the low-decrease group (C1). Similarly, they were less likely to be in these groups than the low-increase group (C4).

**Table 4:** Logistic regression of predictors on school refusal behavior

	high-stable group (C2) vs low-decrease		high-decrease group vs (C3) low-decrease		low-increase group (C4) vs low-decrease		high-decrease group (C3) vs high-stable group		high-stable group (C2) vs low-increase		high-decrease group (C3)vs low-increase	
	group (C1)		group (C1)		group (C1)		(C2)		group(C4)		group(C4)	
	OR	*P*	OR	*P*	OR	*P*	OR	*P*	OR	*P*	OR	*P*
Academic resilience	**0.552**	< **0.001**	**0.535**	< **0.001**	0.346	0.887	0.969	0.974	**0.632**	**0.002**	**0.612**	**0.005**
Family functioning	**0.453**	< **0.001**	**0.446**	< **0.001**	0.649	0.068	0.984	0.937	0.698	0.136	0.687	0.194
Class interpersonal harmony	**0.258**	< **0.001**	**0.242**	< **0.001**	**0.581**	**0.012**	0.941	0.766	**0.443**	< **0.001**	**0.417**	**0.002**

Bold indicates *p* < .05

Regarding the family functioning effect, compared to the low-decrease group (C1), the high-stable group (C2) had an OR value of 0.453 (*p* < 0.001), and the high-decrease group (C3) had an OR value of 0.446 (*p* < 0.001). These results indicated that students with greater family functioning were less likely to be in the high-stable group (C2) or the high-decrease group (C3) than in the low-decrease group (C1).

Regarding the class interpersonal harmony effect, compared to the low-decrease group (C1), the high-stable group (C2) had an OR value of 0.258 (*p* < 0.001), the high-decrease group (C3) had an OR value of 0.242 (*p* < 0.001), and the low-increase group (C4) had an OR value of 0.581 (*p* = 0.012). Compared to the low-increase group (C4), C2 had an OR value of 0.443 (*p* < 0.001), and C3 had an OR value of 0.417 (*p* = 0.002). These results indicated that students with greater interpersonal harmony in class were less likely to be in the high-stable group (C2), the high-decrease group (C3), or the low-increase group (C4) than in the low-decrease group (C1). Additionally, they were less likely to be in the high-stable group (C2) or the high-decrease group (C3) than in the low-increase group (C4).

## Discussion

This study used a longitudinal design to understand the developmental trajectories of school refusal behavior among adolescents. We also examined the predictive effects of individual, family, and school factors on the development of school refusal behavior, as well as whether trajectory membership classes varied depending on the predicted factors. The results provide a more comprehensive understanding of the developmental trajectories of school refusal behavior among adolescents in the Chinese context.

### Heterogeneity of the developmental trajectories of school refusal behavior

On average, school refusal behavior in Chinese adolescents decreased over the three developmental stages assessed in this study; however, the LGMM indicated heterogeneity in the developmental trajectory results. The present study identified four subtypes of school refusal behavior development in Chinese adolescents: Low-decrease group (51.8%), High-stable group (34.0%), High-decrease group (8.0%), and Low-increase group (6.2%). The results indicated that 51.8% of adolescents were in the low‐decrease group, showing a trajectory of school refusal behavior that starts at a low level and decreases over time, compared to the other three groups. This finding is consistent with previous studies on school absences trajectories, such as those by Dräger et al. (2023) and Tunkkari et al. (2025), which have shown that most adolescents display positive developmental trends in school refusal behavior, with low-decrease and low-stable patterns being the most common [23, 24]. However, it is important to note that, in general, school refusal behavior tends to increase significantly during middle school and continues to rise through high school, peaking in the final year [19, 20]. Despite this overall trend, a significant number of students still exhibited low levels of school refusal behavior. The study found that 34% of students were in the high-stable group for school refusal behavior, meaning their initial level of school refusal was high and remained high over time.

This proportion is higher than that found in previous studies on school absences by Benner & Wang (2014), Dräger et al. (2023), and Tunkkari et al. (2025) [21–24], suggesting potential contextual variation in refusal maintenance. The stability of this pattern aligns with trajectory research on chronic school absenteeism. From a developmental psychopathology perspective, early-onset, stable patterns of school refusal may reflect entrenched avoidance behaviors that become self-perpetuating through negative reinforcement. Another group that initially displayed a high level and subsequently showed a sharp decrease to a low level of school refusal behavior, accounting for 8.0%. The high-decrease group indicated that adolescents initially exhibit a high level of school refusal behavior but show a downward trend over time, reflecting a positive developmental pattern. This finding is consistent with prior research on school refusal behavior, such as Benoit et al. (2024), which also identified a similar trajectory [25]. This suggests that while some adolescents may initially struggle with school refusal, their behavior can improve over time, highlighting the potential for positive change and the importance of supportive interventions. The study identified a low-increase group, accounting for 6.2%, in which adolescents started with low levels of school refusal behavior, which then gradually increased over time. This negative developmental pattern is consistent with findings from previous studies by Benner and Wang (2014), Dräger et al. (2023, 2024), and Tunkkari et al. (2025), which also reported similar trajectories [21–24]. This finding mirrors the general trend of school refusal behavior, which tends to increase sharply in middle school and continue to rise through high school, peaking in the final year [19, 20]. This suggests that while some students may initially exhibit low levels of school refusal behavior, their symptoms can worsen over time, underscoring the importance of early identification and intervention.

### Predictors of developmental trajectories of school refusal behavior

The findings revealed that academic resilience, family functioning, and class interpersonal harmony were statistically associated with different developmental trajectories of school refusal behavior. Our findings revealed that higher levels of academic resilience were less frequently observed in the high-stable and high-decrease trajectories of school refusal behavior. This aligns with prior research, such as that by Seçer and Ulaş (2020), which suggests that academic resilience can mitigate multiple risk factors for school refusal behavior [16]. Academic resilience, defined as the ability to achieve academic success despite challenging circumstances [66], serves as a protective factor, helping students maintain psychological well-being and reducing the likelihood of school refusal. Behavior. Similarly, Ingul and Nordahl (2013) emphasized the importance of psychological resilience in reducing school dropout rates among young people [40]. Aslan (2018) further reinforces this viewpoint, suggesting that academic resilience can mitigate the adverse effects of school refusal and related psychological issues on students’ school attachment and attendance [67]. From the perspective of Hobfoll’s (1989) conservation of resources theory [33], academic resilience can be viewed as a psychological resource that enables students to protect and acquire essential resources for managing academic stress, thereby reducing the risk of school refusal behavior. Therefore, cultivating academic resilience is essential for preventing school refusal behavior and promoting positive school attendance and attachment.

Our results showed that adolescents with better family functioning were less likely to be in the high‐stable and high‐decrease groups of school refusal behavior. This is consistent with prior studies [68] that highlight the difficulty of treating school refusal without parental involvement. Empirical evidence indicates that positive family processes can reduce school absence and dropout rates, while negative processes can increase these risks [69]. Cross‐sectional studies have also found that family functioning is significantly lower in families with children who refuse school compared to those without [1]. This finding aligns with the ecological principle of interdependence, which suggests that parents of children with school refusal behavior may have low self-efficacy, thereby influencing their children in multiple ways [2]. Furthermore, according to Social Learning Theory [70], if parents display anxiety or helplessness when facing challenges, children may learn similar responses. Moreover, parents may lack effective strategies to address their children’s school refusal behavior, such as providing emotional support or encouraging school attendance, which can lead to the persistence of the problem [42]. The current study highlights the importance of involving parents in the treatment of school refusal behavior, as parent-related factors are significant risk factors for school refusal [71]. Given the crucial role parents play in the development and maintenance of school refusal behavior, more parent‐targeted strategies are needed to enhance interventions.

Our results indicated that higher levels of class interpersonal harmony were less commonly observed in the high‐stable, high‐decrease, and low‐increase groups trajectories of school refusal behavior. This aligns with Havik et al. (2015), who found a positive link between problematic peer relationships and school refusal [72]. From the viewpoint of classroom peer ecology, interactions among peers and between teachers and students significantly shape individual behavior [73, 74]. Specifically, positive classroom interactions can provide emotional support and a sense of belonging, which may reduce the likelihood of school refusal behavior. On the contrary, problematic relationships can increase the risk of school refusal behavior by creating a stressful or unwelcoming school environment. Moreover, a positive teacher-student relationship and perceived teacher support have been identified as protective factors against school refusal [75]. Thus, enhancing class interpersonal harmony is crucial for preventing the development of school refusal behavior.

### Implications for precision prevention

The identification of distinct developmental trajectories has direct implications for precision prevention. For the low-decrease group, who exhibit consistently low and decreasing refusal levels, universal prevention strategies such as school-wide social-emotional learning programs may suffice to maintain their adaptive trajectories. These programs should include components that reinforce academic resilience, helping students sustain their capacity to cope with everyday academic stressors. In contrast, the high-stable group requires more intensive, multi-systemic interventions. Given their significantly lower baseline levels of academic resilience, family functioning, and class interpersonal harmony, interventions should target all three ecological levels simultaneously. At the individual level, cognitive-behavioral techniques can help these adolescents build academic resilience. At the family level, family systems therapy aimed at improving emotional bonds and problem-solving capacity can provide the external scaffolding these adolescents lack. At the school level, teacher-student relationship mediation can help create a more supportive classroom environment. This multi-pronged approach addresses the self-perpetuating cycle of resource depletion that characterizes this group. For the high-decrease group, who demonstrate positive recovery trajectories despite high initial levels, the priority is consolidation of gains and relapse prevention. Booster sessions should target both individual-level maintenance—sustaining improvements in academic resilience and reinforcing the personal coping strategies that facilitated their recovery—and environmental-level scaffolding, ensuring continued access to supportive school and family resources. This dual-focus approach guards against reverting to avoidance behaviors when adolescents encounter future stressors, particularly during transitions where protective structures may weaken. Most urgently, the low-increase group represents an escalating risk trajectory that warrants early, targeted intervention focused on building academic resilience and environmental-level resources to prevent the escalating pattern of refusal from becoming entrenched.

### Limitations and recommendations for future research

Finally, this study still has some limitations. Firstly, gender, grade level, and school context were not included as covariates in the LGMM, which limits the interpretability of the identified latent classes. Nevertheless, future research should employ multi-group LGMM to test whether trajectory parameters or predictor effects vary systematically by demographic subgroups, thereby strengthening both internal validity and intervention tailoring. Secondly, the 12-month, three-wave design precludes evaluation of longer-term trajectories. We recommend that future studies adopt longer follow-up periods and increase measurement frequency to examine developmental trajectories more comprehensively. Thirdly, the smallest latent classes yield limited statistical power, and findings regarding these groups should be considered exploratory and require replication with larger samples. Fourthly, reliance on self-report measures introduces shared-method variance. Future studies should collect multi-informant data (parent and teacher ratings). Fifthly, data were collected during final examination periods (January and July), which may temporarily elevate school refusal scores. While the magnitude of this seasonality effect is unknown in the present study, future research should employ seasonal-structured designs with assessments at multiple points across the academic year to explicitly model and control for examination-related fluctuations. Sixthly, predictors were assessed only at baseline (T1). Although these constructs are theoretically relatively stable, future research should include repeated measurements to examine how changes in predictors relate to changes in school refusal trajectories. Seventhly, our analysis focused on trajectories of overall school refusal behavior rather than its constituent functional dimensions. Future research should employ parallel process latent growth mixture modeling to examine how the SRAS dimensions co-develop over time. This would enable identification of distinct functional profiles and their differential associations with long-term outcomes, extending the present findings by linking specific refusal functions to targeted intervention strategies.

## Conclusion

Our investigation into heterogeneity in the developmental trajectories of school refusal behavior among Chinese adolescents’ results show that the levels of school refusal behavior remained relatively stable among students in the low-decrease and high-stable groups. In contrast, the students in the high-decrease group showed a significant decrease over time, while those in the low-increase group showed a substantial increase over the same period. Further, academic resilience, family functioning, and class interpersonal harmony were found to significantly affect the developmental trajectory of school refusal behavior among adolescents.

## Data Availability

The datasets generated and/or analyzed during the current study are not publicly available due privacy but are available from the corresponding author upon reasonable request.

## References

[1] Chockalingam M, Skinner K, Melvin G, Yap MBH. Modifiable parent factors associated with child and adolescent school refusal: a systematic review. Child Psychiatry Hum Dev. 2023;54(5):1459–75. 35397716 10.1007/s10578-022-01358-z PMC10435607

[2] Leduc K, Tougas AM, Robert V, Boulanger C. School refusal in youth: a systematic review of ecological factors. Child Psychiatry Hum Dev. 2024;55(4):1044–62. 36422762 10.1007/s10578-022-01469-7 PMC9686247

[3] Kearney C. School refusal behavior in youth: a functional approach to assessment and treatment. American Psychological Association; 2001.

[4] Kearney CA. School absenteeism and school refusal behavior in youth: a contemporary review. Clin Psychol Rev. 2008;28(3):451–71. 17720288 10.1016/j.cpr.2007.07.012

[5] Pina AA, Zerr AA, Gonzales NA, Ortiz CD. Psychosocial interventions for school refusal behavior in children and adolescents. Child Dev Perspect. 2009;3(1):11–20. 20161056 10.1111/j.1750-8606.2008.00070.x PMC2747113

[6] Fernández-Sogorb A, Sanmartín R, Vicent M, Gonzálvez C, Ruiz-Esteban C, García-Fernández JM. School anxiety profiles in Spanish adolescents and their differences in psychopathological symptoms. PLoS ONE. 2022;17(1):e0262280. 35061775 10.1371/journal.pone.0262280 PMC8782359

[7] Fujita J, Aoyama K, Saigusa Y, Miyazaki Y, Aoki Y, Asanuma K, et al. Problematic Internet use and daily difficulties among adolescents with school refusal behaviors: An observational cross-sectional analytical study: An observational cross-sectional analytical study. Medicine (Baltimore). 2022;101(7):e28916. 35363214 10.1097/MD.0000000000028916 PMC9282062

[8] Gonzálvez C, Bacon V, Kearney CA. Systematic and evaluative review of school climate instruments for students, teachers, and parents. Psychol Sch. 2023;60(6):1781–836.

[9] de los Angeles CPL, Elliott S, Gainor J. School refusal: a case‐based exploration of school avoidance. Brown Univ Child Adolesc Behav Lett. 2023;39(9):1–4.

[10] Tekin I, Aydın S. School refusal and anxiety among children and adolescents: a systematic scoping review. New Dir Child Adolesc Dev. 2022;185:43–65. 10.1002/cad.20484 36161758

[11] Heyne D, Sauter FM, Ollendick TH, Van Widenfelt BM, Westenberg PM. Developmentally sensitive cognitive behavioral therapy for adolescent school refusal: rationale and case illustration. Clin Child Fam Psychol Rev. 2014;17(2):191–215. 24338067 10.1007/s10567-013-0160-0

[12] Filippello P, Buzzai C, Messina G, Mafodda AV, Sorrenti L. School refusal in students with low academic performances and specific learning disorder. The role of self-esteem and perceived parental psychological control. Int J Disabil Dev Educ. 2020;67(6):592–607.

[13] Nelemans SA, Hale WW, Branje SJT, Raaijmakers Q, Frijns T, van Lier PAC, et al. Heterogeneity in development of adolescent anxiety disorder symptoms in an 8-year longitudinal community study. Dev Psychopathol. 2014;26(1):181–202. 24229471 10.1017/S0954579413000503

[14] Dembo R, Briones-Robinson R, Barrett K, Winters KC, Schmeidler J, Ungaro R, et al. The mental health, substance use, and delinquency among truant youths in a Brief Intervention project: a longitudinal study. J Emot Behav Disord. 2013;21(3):176–92. 23914129 10.1177/1063426611421006 PMC3728705

[15] Bauducco SV, Tillfors M, Özdemir M, Flink IK, Linton SJ. Too tired for school? The effects of insomnia on absenteeism in adolescence. Sleep Health. 2015;1(3):205–10. 29073441 10.1016/j.sleh.2015.07.007

[16] Seçer İ, Ulaş S. The mediator role of academic resilience in the relationship of anxiety sensitivity, social and adaptive functioning, and school refusal with school attachment in high school students. Front Psychol. 2020;11:557. 32373002 10.3389/fpsyg.2020.00557 PMC7186501

[17] Rocque M, Jennings WG, Piquero AR, Ozkan T, Farrington DP. The importance of school attendance: findings from the Cambridge Study in Delinquent Development on the life-course effects of truancy. Crime Delinq. 2017;63(5):592–612.

[18] Gonzálvez C, Díaz-Herrero Á, Sanmartín R, Vicent M, Pérez-Sánchez AM, García-Fernández JM. Identifying risk profiles of school refusal behavior: differences in social anxiety and family functioning among Spanish adolescents. Int J Environ Res Public Health. 2019;16(19):3731. 31623358 10.3390/ijerph16193731 PMC6801475

[19] Balfanz R, Byrnes V. Chronic absenteeism: summarizing what we know from nationally available data. Baltimore: Johns Hopkins University Center for Social Organization of Schools; 2012.

[20] Ehrlich SB, Gwynne JA, Allensworth EM. Pre-kindergarten attendance matters: early chronic absence patterns and relationships to learning outcomes. Early Child Res Q. 2018;44:136–51.

[21] Benner AD, Wang Y. Shifting attendance trajectories from middle to high school: influences of school transitions and changing school contexts. Dev Psychol. 2014;50(4):1288–301. 24364827 10.1037/a0035366 PMC3981879

[22] Dräger J, Klein M, Sosu E. Trajectories of School Absences and Pupils’ Academic Performance. UK: Report. University of Strathclyde; 2023.

[23] Dräger J, Klein M, Sosu EM. Trajectories of school absences across compulsory schooling and their impact on children’s academic achievement: an analysis based on linked longitudinal survey and school administrative data. PLoS ONE. 2024;19(8):e0306716. 39133716 10.1371/journal.pone.0306716 PMC11318909

[24] Tunkkari M, Kiuru N, Virtanen T, Vasalampi K. Antecedents of developmental trajectories of school absences among adolescents. Scand J Educ Res. 2025;70(3):618–31.

[25] Benoit L, Chan Sock Peng E, Flouriot J, DiGiovanni M, Bonifas N, Rouquette A, et al. Trajectories of school refusal: sequence analysis using retrospective parent reports. Eur Child Adolesc Psychiatry. 2024;33(11):3849–59. 38602549 10.1007/s00787-024-02419-5 PMC11588807

[26] Cicchetti D, Rogosch FA. Equifinality and multifinality in developmental psychopathology. Dev Psychopathol. 1996;8(4):597–600.

[27] Liu L, Gu H, Zhao X, Wang Y. What contributes to the development and maintenance of school refusal in Chinese adolescents: a qualitative study. Front Psychiatry. 2021;12:782605. 34975580 10.3389/fpsyt.2021.782605 PMC8714792

[28] Melvin GA, Heyne D, Gray KM, Hastings RP, Totsika V, Tonge BJ, et al. The kids and teens at school (KiTeS) framework: An inclusive bioecological systems approach to understanding school absenteeism and school attendance problems. Front Educ. 2019;4:61.

[29] Mutlu Ş, Koşan Y. School refusal behaviors in preschool students: Insights from parents, teachers, and school counselors. School Ment Health. 2025;17(4):1445–59.

[30] Wang Y, Gu H, Zhao X, Liu L. Chinese clients’ experiences throughout family therapy for school-refusing adolescents: a multiperspectival interpretative phenomenological analysis. Acta Psychol. 2024;243(104161):104161. 10.1016/j.actpsy.2024.104161 38280349

[31] Xie J, Cheng W, Xu Y, Yao H. Addressing school refusal behaviour in Chinese children and adolescents. Gen Psychiatr. 2025;38(5):e102079. 41000544 10.1136/gpsych-2025-102079 PMC12458648

[32] Bronfenbrenner U. Ecological systems theory. In: Vasta R, editor. Six theories of child development: Revised formulations and current issues. London: Sage Publications; 1992.

[33] Hobfoll SE. Conservation of resources: a new attempt at conceptualizing stress. Am Psychol. 1989;44(3):513–24. 2648906 10.1037//0003-066x.44.3.513

[34] Battistich V, Solomon D, Watson M, Schaps E. Caring school communities. Educ Psychol. 1997;32(3):137–51.

[35] Eccles JS, Roeser RW. School and community influences on human development. In: Bornstein MH, Lamb ME, editors. Developmental science: an advanced textbook. Mahwah, NJ: Lawrence Erlbaum Associates; 2005.

[36] Alva SA. Academic invulnerability among Mexican-American students: the importance of protective resources and appraisals. Hisp J Behav Sci. 1991;13(1):18–34.

[37] Wang MC, Haertel GD, Walberg HJ. Fostering Educational Resilience in Inner-City Schools. In: Walberg O, Reyes M, Weissberg RP, editors. Children and youth: interdisciplinary perspectives. 1997.

[38] Perez W, Espinoza R, Ramos K, Coronado HM, Cortes R. Academic resilience among undocumented Latino students. Hispanic J Behav Sci. 2009;31:149–81.

[39] Martin AJ, Marsh HW. Academic resilience and its psychological and educational correlates: a construct validity approach. Psychol Sch. 2006;43(3):267–81.

[40] Ingul JM, Nordahl HM. Anxiety as a risk factor for school absenteeism: what differentiates anxious school attenders from non-attenders? Ann Gen Psychiatry. 2013;12(1):25. 23886245 10.1186/1744-859X-12-25 PMC3726429

[41] Beavers R, Hampson RB. The Beavers systems model of family functioning. J Fam Ther. 2000;22(2):128–43.

[42] Carless B, Melvin GA, Tonge BJ, Newman LK. The role of parental self-efficacy in adolescent school-refusal. J Fam Psychol. 2015;29(2):162–70. 25642779 10.1037/fam0000050

[43] Bernstein GA, Warren SL, Massie ED, Thuras PD. Family dimensions in anxious–depressed school refusers. J Anxiety Disord. 1999;13(5):513–28. 10600052 10.1016/s0887-6185(99)00021-3

[44] Lyon AR, Cotler S. Multi-systemic intervention for school refusal behavior: integrating approaches across disciplines. Adv Sch Ment Health Promot. 2009;2(1):20–34.

[45] Jiang G. Classroom social-ecological environment study. Wuhan: Huazhong Normal University Press; 2002.

[46] Jiang G. Classroom environment in primary and secondary schools: structure and measurement. Psychol Sci. 2004;27:839–43.

[47] Chen X, Li B, Li Z. Mother-child relationship,social behavior and peer acceptance in Chinese children. Acta Psychol Sin. 1995;27:329–36.

[48] Chen BB, Li D. Student perceived interpersonal harmony in class and its relationship with social behavior. Psychol Dev Educ. 2009;25(2):41–6.

[49] Buyse E, Verschueren K, Doumen S, Van J D, Maes F. Classroom problem behavior and teacher-child relationships in kindergarten: the moderating role of classroom climate. J Sch Psychol. 2008;46(4):367–91. 19083364 10.1016/j.jsp.2007.06.009

[50] Liu Y, Li X, Chen L, Qu Z. Perceived positive teacher-student relationship as a protective factor for Chinese left-behind children’s emotional and behavioural adjustment. Int J Psychol. 2015;50(5):354–62. 25410645 10.1002/ijop.12112

[51] Hu H, Gao J, Jiang H, Jiang H, Guo S, Chen K, et al. A comparative study of behavior problems among left-behind children, migrant children and local children. Int J Environ Res Public Health. 2018;15(4):655. 29614783 10.3390/ijerph15040655 PMC5923697

[52] Rudasill KM, Rjt G, Stipanovic N, Taylor JE. A longitudinal study of student– teacher relationship quality, difficult temperament, and risky behavior from childhood to early adolescence. J Sch Psychol. 2010;48(5):389–412. 20728689 10.1016/j.jsp.2010.05.001

[53] Silver RB, Measelle JR, Armstrong JM, Essex MJ. Trajectories of classroom externalizing behavior: contributions of child characteristics, family characteristics, and the teacher–child relationship during the school transition. J Sch Psychol. 2005;43(1):39–60.

[54] Li D, Zong L, Liu J. The relationship between externalizing behavior problem and collective moral emotion and responsibility: the moderate effects of class climate. Acta Psychol Sin. 2013;45(9):1015–25.

[55] Olivier E, Morin AJS, Langlois J, Tardif-Grenier K, Archambault I. Internalizing and externalizing behavior problems and student engagement in elementary and secondary school students. J Youth Adolesc. 2020;49(11):2327–46. 32710241 10.1007/s10964-020-01295-x

[56] Kearney CA. Identifying the function of school refusal behavior: a revision of the School Refusal Assessment Scale. J Psychopathol Behav Assess. 2002;24(4):235–45.

[57] Martin AJ, Marsh HW. Academic buoyancy: towards an understanding of students’ everyday academic resilience. J Sch Psychol. 2008;46(1):53–83. 19083351 10.1016/j.jsp.2007.01.002

[58] Sun W. Effects of everyday academic resilience and academic engagement on students’ performance in high school. Changchun: Northeast Normal University Press; 2009.

[59] Smilkstein G. The family APGAR: a proposal for a family function test and its use by physicians. J Fam Pract. 1978;6(6):1231–9. 660126

[60] Lv F, Gu Y. Family APGAR questionnaire and its clinical application. Foreign Med. 1995;11:56–9.

[61] Guo B. Effects of classroom climate on children's social behaviors and their relations to school adjustment in rural China. PhD Thesis. The Chinese University of Hong Kong, Hong Kong; 2004.

[62] Muthén B, Asparouhov T. Growth mixture modeling: Analysis with non-Gaussian random effects. Longitudinal data analysis. 2008;143–165.

[63] Muthén LK, Muthén BO. Mplus user’ s guide. 8th ed. Los Angeles, CA: Muthén & Muthén; 2017.

[64] Wang M, Bi X, Ye H. Growth mixture modeling: a method for describing specific class growth trajectory. Sociol Stud. 2014;29(4):220–41.

[65] Nylund KL, Asparouhov T, Muthén BO. Deciding on the number of classes in latent class analysis and growth mixture modeling: a Monte Carlo simulation study. Struct Equ Modeling. 2007;14(4):535–69.

[66] Rudd G, Meissel K, Meyer F. Measuring academic resilience in quantitative research: a systematic review of the literature. Educ Res Rev. 2021;34:100402.

[67] Aslan M. The Investigation of the Relationship Between Anxiety Sensitivity and School Refusal on the Group of Students Aged 8–14 Years. Master’ s Thesis. University of Otago, Dunedin; 2018.

[68] Elliott JG, Place M. Practitioner review: school refusal: developments in conceptualisation and treatment since 2000. J Child Psychol Psychiatry. 2019;60(1):4–15. 29197106 10.1111/jcpp.12848

[69] Marlow SA, Rehman N. The relationship between family processes and school absenteeism and dropout: a meta-analysis. Educ Dev Psychol. 2021;38(1):3–23.

[70] Bandura A. Self-efficacy: toward a unifying theory of behavioral change. Psychol Rev. 1977;84(2):191–215. 847061 10.1037//0033-295x.84.2.191

[71] Eroglu M, Mentese Babayigit T, Bilgen Ulgar S, et al. School refusal and determinants: Parental psychopathology, family functioning, attachment and temperament. Psychol Sch. 2025;62(3):853–63.

[72] Havik T, Bru E, Ertesvåg SK. Assessing reasons for school non-attendance. Scand J Educ Res. 2015;59(3):316–36.

[73] Gest SD, Rodkin PC. Teaching practices and elementary classroom peer ecologies. J Appl Dev Psychol. 2011;32(5):288–96.

[74] Hendrickx MM, Mainhard MT, Boor-Klip HJ, Cillessen AH, Brekelmans M. Social dynamics in the classroom: teacher support and conflict and the peer ecology. Teach Teach Educ. 2016;53:30–40.

[75] Ulaş S, Seçer İ. A systematic review of school refusal. Curr Psychol. 2024;43(21):19407–22.

